# Comparison of large-scale human brain functional and anatomical networks in schizophrenia

**DOI:** 10.1016/j.nicl.2017.05.007

**Published:** 2017-05-14

**Authors:** Brent G. Nelson, Danielle S. Bassett, Jazmin Camchong, Edward T. Bullmore, Kelvin O. Lim

**Affiliations:** a Department of Psychiatry, University of Minnesota, Minneapolis, MN 55454, USA; b Department of Physics, University of California, Santa Barbara, CA 93106, USA; c Sage Center for the Study of the Mind, University of California, Santa Barbara, CA 93106, USA; d Department of Bioengineering, University of Pennsylvania, Philadelphia, PA 19104, USA; e Behavioural & Clinical Neuroscience Institute, University of Cambridge, Cambridge, UK; f Cambridgeshire & Peterborough NHS Foundation Trust, Cambridge, UK; g GlaxoSmithKline R&D, Cambridge, UK

**Keywords:** Network, Connectivity, Schizophrenia

## Abstract

Schizophrenia is a disease with disruptions in thought, emotion, and behavior. The dysconnectivity hypothesis suggests these disruptions are due to aberrant brain connectivity. Many studies have identified connectivity differences but few have been able to unify gray and white matter findings into one model. Here we develop an extension of the Network-Based Statistic (NBS) called NBSm (Multimodal Network-based statistic) to compare functional and anatomical networks in schizophrenia. Structural, resting functional, and diffusion magnetic resonance imaging data were collected from 29 chronic patients with schizophrenia and 29 healthy controls. Images were preprocessed, and average time courses were extracted for 90 regions of interest (ROI). Functional connectivity matrices were estimated by pairwise correlations between wavelet coefficients of ROI time series. Following diffusion tractography, anatomical connectivity matrices were estimated by white matter streamline counts between each pair of ROIs. Global and regional strength were calculated for each modality. NBSm was used to find significant overlap between functional and anatomical components that distinguished health from schizophrenia. Global strength was decreased in patients in both functional and anatomical networks. Regional strength was decreased in all regions in functional networks and only one region in anatomical networks. NBSm identified a distinguishing functional component consisting of 46 nodes with 113 links (p < 0.001), a distinguishing anatomical component with 47 nodes and 50 links (p = 0.002), and a distinguishing intermodal component with 26 nodes (p < 0.001). NBSm is a powerful technique for understanding network-based group differences present in both anatomical and functional data. In light of the dysconnectivity hypothesis, these results provide compelling evidence for the presence of significant overlapping anatomical and functional disruption in people with schizophrenia.

## Highlights

• A novel method for the unified analysis of functional and anatomical networks in schizophrenia is proposed.
• The methodology extends existing networkbased analyses to test brain network differences across imaging modalities.
• A fundamental disrupted network was found in those with schizophrenia when compared to healthy, agematched controls.

## 1 Introduction

Schizophrenia is characterized by a host of observable abnormalities in integrated thought, emotion, and behavior. Lack of integration is hypothesized to stem from multiple abnormalities in the underlying brain circuitry, collectively referred to as *dysconnectivity*. The hypothesis that dysconnectivity drives psychiatric symptoms in schizophrenia is supported by neuroimaging studies utilizing both functional magnetic resonance imaging (fMRI) and diffusion tensor imaging (Friston, 1998, Volkow et al., 1988, Weinberger et al., 1992). Dysconnectivity can manifest separately as either differences in coherent brain activity (functional connectivity) or brain wiring (anatomical connectivity) (Camchong et al., 2011, Skudlarski et al., 2010).

While dysconnectivity can accompany many different disease states, the specific connectivity abnormalities identified in schizophrenia patients remain far from understood. Anatomical connectivity estimated from white matter tracts is altered in schizophrenia in a range of disparate cortical structures including frontal regions (Kong et al., 2011), thalamo-frontal connections (Oh et al., 2009), temporal-frontal connections (van den Heuvel et al., 2010), and temporal tracts (Phillips et al., 2009). Alterations in functional connectivity have similarly been identified across a range of brain states, affecting the default mode network (DMN) at rest (Whitfield-Gabrieli et al., 2009, Woodward et al., 2011), multiple cognitive control networks (Repovs et al., 2011), and several independent brain regions (Zhou et al., 2007). However, the focus of much of this previous work has been limited to a few selected regions or tracts and to a single imaging modality, potentially hampering a broader understanding of a distributed pathophysiology.

Anatomical and functional connectivity are inherently related (Schneider et al., 2007, Skudlarski et al., 2010). Evidence suggests that anatomical connectivity patterns underlie resting-state and task-based functional connectivity patterns (Hermundstad et al., 2013, Honey et al., 2009, Teipel et al., 2010). A simultaneous examination of whole-brain anatomical and functional connectivity in a single cohort is necessary for a more comprehensive understanding of putative alterations in brain architecture that underlie abnormal cognition and behavior in schizophrenia.

Multimodal techniques in depression (Hermundstad et al., 2013) and schizophrenia (Jeong et al., 2009, Pomarol-Clotet et al., 2010, Skudlarski et al., 2010) have provided a holistic characterization of these diseases inaccessible from either modality alone. However, the translational impact of these studies has likely been hampered by inconsistent results. Several whole-brain connectivity studies have identified anatomical and functional abnormalities in frontal regions in schizophrenia patients: two studies examining the resting state and one study examining task-based states (Camchong et al., 2011, Jeong et al., 2009, Pomarol-Clotet et al., 2010). Zhou et al. (2008) report converging anatomical and functional connectivity abnormalities between the hippocampus and the rest of the brain in people with schizophrenia (Zhou et al., 2008). The use of divergent analysis methods makes unifying these findings difficult. Skudlarski et al. (2010) described one method to address this challenge and report convergent findings across multiple imaging modalities.

An important challenge for multimodal studies is the identification of methodological approaches capable of unifying disparate types of data. Networks provide a mathematical framework to describe interactions between system entities and can therefore be particularly useful in this context. A powerful and versatile approach, network science can be used to examine the relationships between entities as varied as routers in the internet, friends in a social network, or regions in the human brain (Honey et al., 2007). The network being studied is defined as a graph in which the system's components are represented as nodes in the graph and interactions between the system's components are represented as edges in the graph. This information is encoded in a mathematical data structure called a connectivity matrix, which is composed of rows, columns, and cells, similar to a spreadsheet. Rows and columns represent nodes and cell values represent edges connecting these nodes.

Graph theory has been applied with increasing success to neuroimaging data (Bullmore and Sporns, 2009, Bullmore and Bassett, 2011). Specifically, it is used to quantify the organization of the brain and estimate its information-processing efficiency. In addition to facilitating the examination of healthy brain function, graph theory also provides a means to examine altered brain function in psychiatric disease (Fornito et al., 2012). As a unified approach, network theory can be applied to both anatomical and functional data to derive estimates of connectivity. Its application to schizophrenia in particular has uncovered decreased functional connectivity with increased variation between frontal and temporal regions (Bassett et al., 2012, Lynall et al., 2010, Van den Heuvel et al., 2010, Yu et al., 2011, Zalesky et al., 2010). Its application to diffusion imaging has uncovered anatomical connectivity differences between regions including medial frontal, parietal/occipital, and the left temporal lobe (Zalesky et al., 2011).

Statistical inference of significant group differences in network diagnostics of brain connectivity has remained challenging. A common strategy to construct a graph is to threshold a connectivity matrix to retain only the very strongest and/or most statistically significant edges (Bullmore and Bassett, 2011). To compare graphs between two groups, the threshold is often chosen for each matrix independently in order to ensure that all networks, irrespective of group, contain the same number of edges (van Wijk et al., 2010). However, it has been noted that for very stringent thresholds, networks derived from one population can fragment (some nodes become completely disconnected from the graph, having no remaining edges) while networks derived from a second population can remain intact. This phenomenon has been reported to occur in resting state fMRI data acquired from people with schizophrenia (Bassett et al., 2012). In this context, fragmentation has been linked to underlying network developmental abnormalities (van den Berg et al., 2012). Comparing fragmented and non-fragmented networks is problematic because network diagnostic values are highly dependent on the number of nodes present in the network (Bassett et al., 2012, Van Wijk et al., 2010). In addition to the challenges of the differential fragmentation processes, this common approach also focuses on only the strongest set of edges although recent evidence suggests that in fact weakly connected portions of the network might be particularly important in distinguishing healthy and diseased resting state function in schizophrenia (Bassett et al., 2012), and uncovering changes in network organization that underlie individual differences in cognitive function (Cole et al., 2012, Santarnecchi et al., 2014). In combination, these methodological factors underscore the potential benefits of developing alternative approaches.

A recently developed methodology known as the Network-Based Statistic (NBS) can be used to circumvent several issues that accompany the comparison of networks extracted from thresholding procedures (Zalesky et al., 2010). NBS uses a permutation-based approach to select sub-networks (also known as network components) formed by edges whose weights are significantly different between the two groups. Importantly, these edges are identified irrespective of whether their weights are strong or weak. A benefit of this technique is that it is safeguarded against the multiple comparisons problem that one faces in the pairwise comparison of all edges between the two groups. While both Bonferroni and false discovery rate (FDR) corrections can be employed, they are arguably overly-conservative for the set of inherently dependent variables that make up connectivity matrices (Zalesky et al., 2010).

Here we apply NBS to both functional and anatomical data to identify connectivity abnormalities. We further develop an extension of NBS for use in the simultaneous examination of multimodal data, which we call NBS_m_. This method allows us to statistically test for overlapping regions of dysconnectivity in any two groups, facilitating the identification of an overlapping or “intermodal” set. We apply this method to a clinical population of 29 people with schizophrenia and 29 age- and sex-matched controls. We hypothesize that a subset of regions identified as locations of either functional or anatomical dysconnectivity in schizophrenia will show statistically significant overlap when examined from the perspective of distinguishing sub-networks identified using NBS_m_.

## 2 Methods and materials

### 2.1 Participants

Data from 29 participants with chronic schizophrenia (11 females; age 41.3 ± 9.3 (SD); 5 left-handed) and 29 healthy participants (11 females; age 41.1 ± 10.6 (SD); 2 left-handed) were included in this analysis. All participants provided written informed consent and received payment for the time they spent participating. The consent process and all procedures were reviewed and approved by the institutional review board (IRB) at the University of Minnesota prior to initiating studies. Schizophrenia patients were diagnosed with the Structured Clinical Interview for *DSM-IV*. Out of the 29 chronic schizophrenia patients: 16 were taking 1 atypical antipsychotic, 8 were taking 2 atypical antipsychotics, 1 was taking 1 typical antipsychotic, 1 was taking 1 atypical and 1 typical antipsychotic, 1 was taking 2 atypical and 1 atypical antipsychotics, and 2 were not taking any antipsychotic. Participants were excluded if they fulfilled the criteria for Alcohol or Substance Abuse or Dependence described in the *Diagnostic and Statistical Manual of Mental Disorder, Fourth Edition* within 3 months prior to scanning, significant medical illness, or head injury resulting in loss of consciousness exceeding 30 min. Education and occupational status were determined with the Socioeconomic Status questionnaire. See (Camchong et al., 2011) for a detailed breakdown of educational and socioeconomic status. Groups were matched by levels of education (*t*(55) = 0.06, p = 0.952) and occupational status (*t*(55) = 1.191, p = 0.239) of primary caregivers. Healthy participants, however, had significantly higher levels of education (*t*(55) = 5.01, p < 0.001) and occupational status (*t*(55) = 5.78, p < 0.001). See Bassett et al. (2012) for a detailed analysis of the whole-brain resting state connectivity in this population.

### 2.2 Imaging

Structural (sMRI), functional (fMRI), and diffusion weighted (DTI) magnetic resonance images were acquired for all participants on a Siemens 3.0 T Trio scanner (Erlangen, Germany). All images were acquired in a single scanning session.

The resting state fMRI scan lasted for approximately 6 min. Participants were given instructions to remain still, keep their eyes closed, and remain awake. The scan utilized a gradient-echo echo-planar imaging (EPI) sequence of 180 volumes, a TR (repetition time) of 2 s, TE (echo time) of 30 ms, flip angle of 90° and consisted of 34 continuous AC-PC aligned axial slices with a voxel size of 3.4 × 3.4 × 4.0 mm and an acquisition matrix size of 64 × 64 × 34. Participants were debriefed at the end of the scan to find out whether they fell asleep.

DTI images were acquired axially using a dual spin echo, single-shot pulsed-gradient echo-planar imaging (EPI) technique. The sequence included a TR of 8.3 s, TE of 86 ms, *b*-value of 1000 s/mm^2^, 2 averages, and consisted of 64 slices with a voxel size of 2 × 2 × 2 mm, a field of view of 256 mm, a skip of 0 mm, and 30 non-collinear directions.

To measure brain structure, we employed a high-res T1 weighted anatomical image that utilized a magnetization prepared rapid gradient-echo sequence. A field map was also generated and included a TR of 300 ms, TE of 1.91 ms/4.37 ms, a flip angle of 55°, and a voxel size of 3.4 × 3.4 × 4.0 mm.

### 2.3 Pre-processing

All images were pre-processed to reduce noise from motion and inhomogeneity in the magnetic field, and to produce connectivity matrices for later analysis. All fMRI and DTI images were registered to high resolution anatomical space.

Careful attention was paid to motion correction since subtle movements have been shown to introduce spurious correlations between time series (Power et al., 2012, Satterthwaite et al., 2012). Importantly, the two groups had similar mean root mean square (RMS) motion parameters for the fMRI acquisitions: two-sample *t*-tests of mean RMS translational and angular movement were not significant (p = 0.14 and p = 0.12, respectively).

fMRI images were processed by removing the first 3 volumes for magnetization stabilization, correcting motion artifacts using FSL, correcting for geometric distortion, correcting for slice-timing effects, and removing non-brain elements such as skull, meninges, and dura. Spatial smoothing, using a 6 mm full-width half-max kernel, grand mean and intensity normalization, and high-pass temporal filtering were also applied to images. fMRI images were denoised using probabilistic independent component analysis (PICA) using a tool in FSL (http://www.fmrib.ox.ac.uk/fsl/), which was based on the procedure established by Kelly et al. (2010). The goal of this procedure was to remove noise components from head motion scanner artifacts, and physiological noise as described by Camchong et al. (2011) and in the MELODIC manual (http://www.fmrib.ox.ac.uk/fslcourse/lectures/melodic.pdf). Linear regression was performed on each voxel to account for effects of individual subject motion by removing 6 motion parameter time courses. After registration to high resolution anatomical space, fMRI images were then registered to Montreal Neurological Institute (MNI) standard space (MNI-152 brain).

Average time series were extracted for 90 cortical and subcortical regions of interest (ROIs) based on the Automated Anatomical Labeling (AAL) atlas (Tzourio-Mazoyer et al., 2002). ROIs from the cerebellum and vermis were not included in this analysis. We used a maximum overlap discrete wavelet transform to extract time series data from specific frequency bands. We chose the Daubechies 4 wavelet and extracted level 2 wavelet coefficients for each ROI, corresponding to a frequency range of 0.060–0.125 Hz. We constructed a functional connectivity matrix by computing Pearson correlation coefficients for each pair of ROI time series.

DTI images were corrected for distortion due to eddy currents and head motion (using a 6 degrees of freedom linear registration) and were further corrected for geometric distortion using field maps generated during the structural scan. The AAL atlas was then transformed to native space for each subject using a non-linear registration (FNIRT; http://www.fmrib.ox.ac.uk/fsl/fnirt). White matter tractography files were generated by a FACT-based, DTI algorithm using the TrackVis Diffusion Toolkit (trackviz.org); parameters included a 35 degree angle threshold, 0.1 step threshold, and voxel center seed location using one seed per voxel, with analysis applied to the whole brain. This algorithm was applied to the whole brain according to the TrackVis Diffusion Toolkit guidelines. We constructed an anatomical connectivity matrix by counting the number of white matter streamlines linking each pair of ROIs, in a similar manner to that reported in (Zhang et al., 2015).

### 2.4 Brain size analysis

Structural T1 volumetric data were processed with FreeSurfer. Using the FreeSurfer BrainSegNotVent variable, we compared brain size between the two groups with age and gender as covariates. No significant group difference was found.

### 2.5 Bivariate analysis

As described above, for each data type a 90 × 90 (N × N) connectivity matrix was generated. This matrix describes the (functional or anatomical) connectivity between pairs of brain regions (4005 pairs in total). We then calculated the average connectivity between each region and all 89 other regions. This measure of connectivity is referred to as *regional strength*. Specifically, the strength of node (region) *i* is defined as the mean value of the *i*th column of the connectivity matrix. Overall brain connectivity, referred to as the *global strength*, was then calculated. Specifically, the global strength was calculated by averaging all regional strengths into one value. Group comparisons were made for both anatomical and functional data types and for both regional and global strength measures. Comparisons were calculated via an independent two sample *t*-statistic. This was calculated as the mean group difference divided by the pooled standard deviation. Significance was tested by permutation testing (Nichols and Holmes, 2002). Ten million random permutations were performed for regional and global strength for both anatomical and functional data. For each permutation, group labels were randomized and a *t*-statistic was calculated. This *t*-statistic extracted from the permuted data was then included as a member of the null distribution. The position of the true *t*-statistic was then located in the null distribution to determine a p-value. The p-value was calculated by dividing the count of null *t*-statistic values larger than the true *t*-statistic by the total number of permutations. Control for Type-I (false positive) errors that accompany multiple comparisons, specifically for the regional analysis, was then performed using a false discovery rate (FDR) correction (Storey, 2003).

### 2.6 Network analysis

Here we used the Network-Based Statistic (NBS) developed by Zalesky et al. (2010) to determine group differences in connectivity within each imaging modality. In a second-level analysis, we evaluated the overlap of significantly different, connected ROIs between modalities using a multimodal extension NBS_m_ which we develop for this study.

#### 2.6.1 Intramodal differences (NBS)

**Fig. 1:**
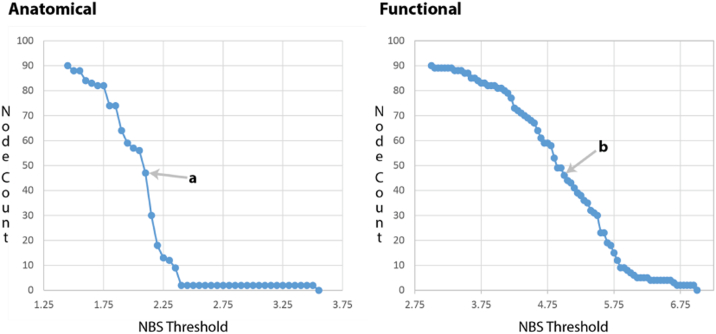
Visualization of the relationship between NBS threshold and the number of nodes present in the largest intramodal component for both anatomical and functional data sets. Labels a and b represent the specific threshold chosen for each modality in the NBSm analysis. They were chosen to select NBS components of similar size involving approximately 50% of total network nodes. Label a represents a threshold of 2.10 with a network node count of 47. Label b represents a threshold of 5.00 with a network node count of 46.

Subjects were labeled as proband or control and group differences between matrix elements were identified by generating a *t*-statistic for each element in the connectivity matrices. In this way, we constructed a *t*-matrix representing group differences in connection strengths for each modality. The *t*-matrix for each modality was thresholded to retain information only for matrix elements with the largest group differences. An initial data exploration was performed to find the *t*-threshold for each data modality. A distribution of node count (of the largest component) versus *t*-threshold was generated for each modality over a wide range of possible *t*-thresholds. Since the anatomical imaging had a lower distribution of *t*-thresholds it was used to choose a maximal node count provided by a *t*-threshold ≥ 2.00 (Fig. 1) and resulted in a *t*-threshold of 2.10. Based on node count this resulted in the selection of a functional *t*-threshold of 5.00. This method provided similarly sized components even within very different imaging modalities.

Once a *t*-threshold was chosen for each modality, each *t*-matrix was thresholded and subnetworks were identified. The largest intramodal component was defined as the subnetwork of the modality-specific *t*-matrix with the largest total number of edges. In short, we use the size of the largest connected component as a statistic; noting that this sub-graph isolates significant network-based differences between the two groups. In other words, we are testing against the null hypothesis that the size of a sub-graph of group differences is larger than expected by chance.

#### 2.6.2 NBS significance testing

**Fig. 2:**
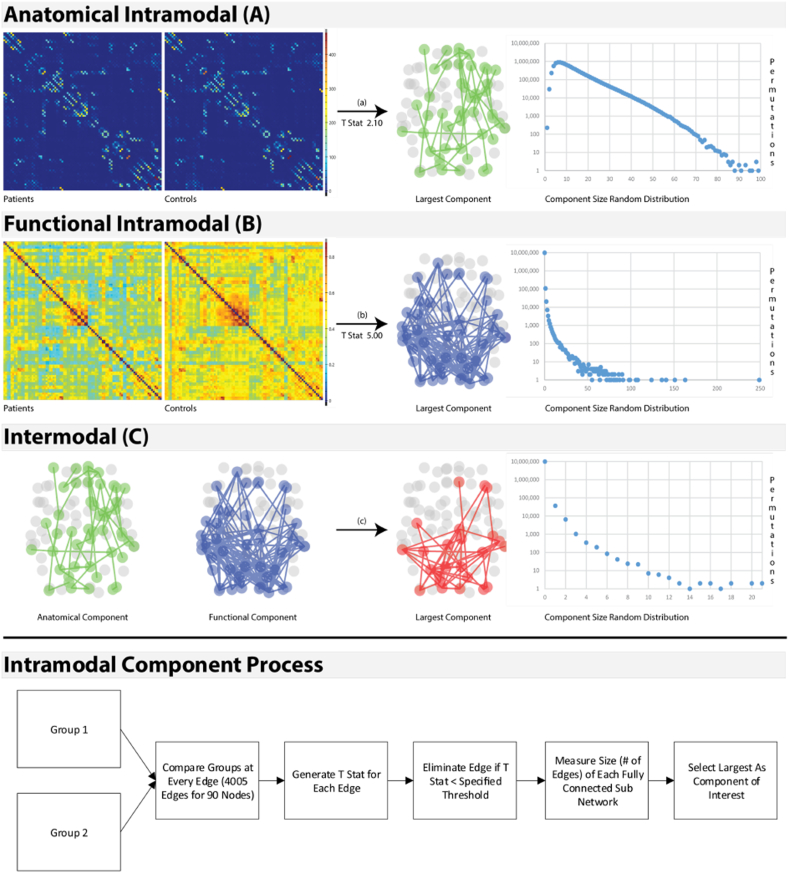
Visualization of the processes used to generate both the intramodal components and intermodal set. Sub-figures A and B refer to the modality-specific intramodal component process using the T stats specified. The largest modality-specific components are shown along with the random distribution generated to compute significance. Sub-figure C shows the computed overlap with the random distribution generated to compute significance.

The significance of intra-modal components was tested using a permutation testing technique (Fig. 2). The intra-modal component generation process was repeated for each modality with random reassignment of subject labels but with maintenance of original group sizes. For each random permutation, the maximum component size was calculated and stored in a null intra-modal distribution. After 10 million permutations, the size of the actual intra-modal components was compared to the null size distribution extracted from the permuted data sets. The p-value for significance in comparison to the null hypothesis was calculated by dividing the total number null model components larger than the actual component by the total number of permutations performed.

#### 2.6.3 Intermodal overlap (NBS_m_)

After determining the intra-modal component for each modality, we investigated the anatomical locations of regions in each component separately and defined an “intermodal overlap” set to be the subset of nodes present in both intra-modal components.

#### 2.6.4 NBS_m_ significance testing

The significance of the intermodal set was determined using a set membership procedure. With each permutation, the number of overlapping ROIs (i.e., those nodes present in both intra-modal components) was determined. The size of the intermodal overlap set and identity of the ROIs in this group were recorded. A p-value for the significance of the size of the intermodal overlap set was generated by counting the number of permuted data sets in which the size of the intermodal overlap set was larger than the actual size and dividing this number by the number of permutations. A p-value for the significance of the presence of each ROI in the intermodal overlap set was determined by counting the number of permutations in which the ROI was identified as a member of the intermodal overlap set and dividing this number by the number of permutations.

## 3 Results

### 3.1 Global strength

**Fig. 3:**
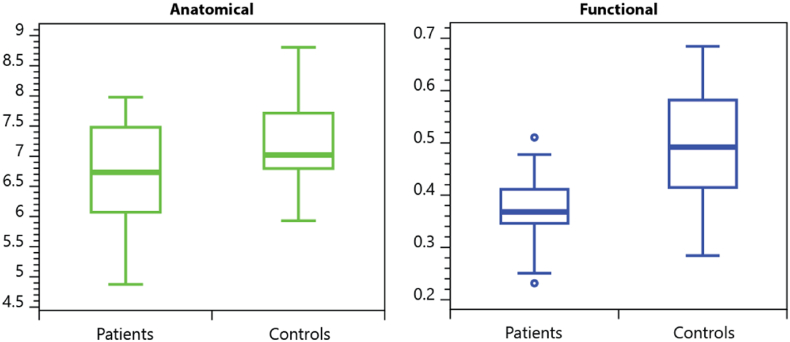
Group differences in global strength of connectivity in anatomical (left) and functional (right) brain networks.

Patients showed significantly lower global strength for both anatomical and functional network data (Fig. 3). In the anatomical data, global strength was weaker by 0.451 (p < 0.001); this corresponds to a smaller number of white matter streamlines linking brain areas. In the functional data, global strength was weaker in patients by 0.113 (p < 0.001). This indicates weaker functional connectivity between brain areas when patients are compared to controls.

### 3.2 Regional strength

Regional strength varied significantly between the two groups for both modalities. Both the anatomical and functional data in people with schizophrenia displayed significantly lower regional strength than controls (p < 0.05, FDR corrected) but differed in the number of significant regions. In the anatomical data, only one region showed patients having lower strength (superior medial frontal cortex in the left hemisphere), whereas in the functional data, all 90 regions showed a group difference in strength.

### 3.3 Functional component

**Fig. 4:**
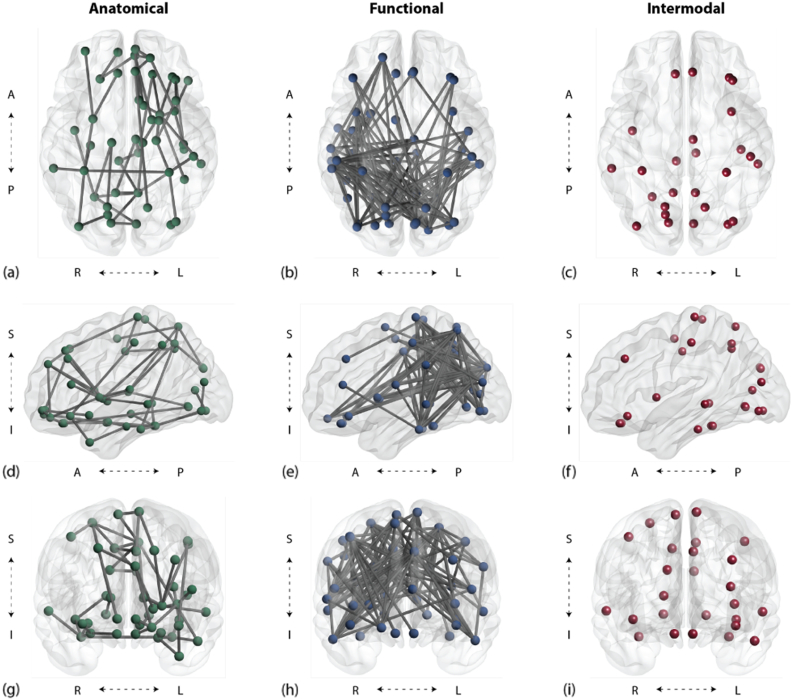
Axial, sagittal, and coronal views of modality-specific (NBS) components and intermodal (NBSm) set. All components are significant at p < 0.001. Colored circles and lines indicate nodes and edges respectively that have been identified in the structural (left), functional (middle), and intermodal (right) components; gray circles indicate nodes that were not present in the significant components.

We applied NBS to the functional connectivity matrices of both groups estimated from the resting-state BOLD signal. We identified an intra-modal component, distinguishing group differences in functional connectivity, which consisted of 46 nodes linked by 113 edges (Table 1). The size of this component was significantly larger than expected under the null hypothesis (p < 0.001). Subsequent analysis showed that patients had weaker connectivity than controls (defined as the average correlation between intra-modal network nodes): with a difference between groups of approximately − 0.224. This component was globally distributed but consisted of more areas of difference in the posterior right cortices (Fig. 4).

**Table 1:** AAL atlas nodes found to be significant in intermodal data sets (top), functional data sets (middle), and anatomical data sets (bottom). Functional and anatomical nodes listed are in addition to their shared intermodal nodes (present in both anatomical and functional).

*Intermodal nodes (present in both anatomical and functional) – 26 nodes*
Temporal Mid R, Precuneus R, Lingual L, Lingual R, Rectus L, Precuneus L, Calcarine R, Precentral R, Paracentral Lobule L, Occipital Mid L, Fusiform R, Parietal Sup R, Paracentral Lobule R, Cuneus L, Cuneus R, Rectus R, Temporal Mid L, Insula L, Temporal Inf L, Frontal Inf Orb L, Cingulum Mid L, Frontal Mid L, Postcentral L, Fusiform L, Occipital Inf R, Occipital Inf L

*Unique functional nodes – 21 nodes*
Frontal Sup L, Frontal Sup R, Frontal Sup Orb L, Frontal Mid Orb R, Frontal Inf Tri L, Olfactory L, Frontal Sup Medial L, Frontal Med Orb L, Frontal Med Orb R, Hippocampus R, ParaHippocampal L, Amygdala L, Amygdala R, Parietal Inf L, Caudate L, Putamen L, Pallidum L, Thalamus L, Thalamus R, Heschl L, Temporal Pole Mid L

*Unique anatomical nodes – 20 nodes*
Frontal Sup Orb R, Frontal Inf Orb R, Rolandic Oper L, Rolandic Oper R, Supp Motor Area R, Insula R, Cingulum Ant L, Calcarine L, Occipital Sup L, Occipital Sup R, Occipital Mid R, Postcentral R, Parietal Sup L, Parietal Inf R, SupraMarginal L, SupraMarginal R, Angular R, Putamen R, Temporal Sup R, Temporal Inf R

### 3.4 Anatomical component

We next applied NBS to the anatomical connectivity matrices estimated via diffusion tensor imaging (DTI). We identified an intra-modal component, distinguishing group differences in anatomical connectivity, which consisted of 47 nodes linked by 50 edges (Table 1). The size of this component was significantly larger than expected under the null hypothesis (p = 0.002). We found that patients had weaker connectivity than controls (defined as the average number of tracts between network nodes); the average difference between groups was approximately − 13.10 streamlines for each region. The 47 nodes of this component were distributed over the medial aspect of the brain with a higher proportion located in the left hemisphere than in the right hemisphere (Fig. 4).

### 3.5 Intermodal set

We used NBS_m_ to identify the intersection between statistically different functional and anatomical connectivity patterns in the two groups. We found that 26 nodes were shared between the functional component and the anatomical component identified in the previous section, together forming the intermodal overlap set. These overlapping nodes are listed in Table 1.

The inter-modal overlap set contained significantly more nodes than expected under the null hypothesis of no significant overlap between functional and anatomical connectivity differences (p < 0.001). These nodes were distributed over the medial, left, and posterior aspects of the brain (Fig. 4). In 10 million permutations, the largest number of nodes identified as members of an intermodal overlap set in the permuted data sets was 11, a number much smaller than the observed number of 26 in the true inter-modal overlap component. In fact, approximately 90% of all permutations showed zero overlap between intra-modal component nodes. Further, a permutation analysis was performed to determine the probability of each individual node being present in the overlap group (see Methods and materials). Each node was found to be present at a significance level of at least p < 0.001.

## 4 Discussion

Evidence suggests that differences in brain architecture and function between those with and without schizophrenia are vast (Camchong et al., 2011, Skudlarski et al., 2010). A range of methodologies have been used to identify these differences both in anatomy (using diffusion imaging and morphometric variation) and function (using fMRI, EEG, or MEG). However, the interplay between anatomical and functional abnormalities is far from understood. Powerful techniques have been developed to examine both anatomical and functional connectivity but few studies have been able to unify the findings within these separate modalities. The recent development of graph theoretical methods for the statistical evaluation of neuroimaging data has facilitated the examination of anatomical and functional connectivity under a common mathematical framework (Bullmore and Sporns, 2009, Bullmore and Bassett, 2011). While high-level information about dysconnectivity can be gathered by examining connectivity at a global or regional level, many of the subtleties are lost due to restrictive statistical corrections. Further, by using novel statistical methods – including group comparisons that employ the Network-Based Statistic and significance testing utilizing non-parametric permutation-based techniques – a principled framework is now available to find a single significant nodal set that summarizes common differences across imaging modalities.

We examined measures of global and regional strength and identified decreased connectivity in people with schizophrenia in both anatomical and functional data. This hypoconnectivity is consistent with results reported in previous studies (Bassett et al., 2012, Lynall et al., 2010). We also identified decreases in regional strength in both anatomical and functional networks, suggesting a regional specificity of disease-related alterations in network structure which is consistent with the prior literature (Lynall et al., 2010, Van den Heuvel et al., 2010). Moreover, the complex pattern of decreases in connectivity supports the dysconnectivity hypothesis (Friston, 1998) of schizophrenia.

Gross descriptors of anatomical and functional connectivity, such as global and regional strength, are limited because they are averages and do not utilize information contained in specific network connections; they therefore cannot be used to identify how the nodal differences are related to one another. An additional difficulty of regional analysis is that nodal measures of different modalities are difficult to compare statistically to one another due to wide differences in variance and modality-specific trends. These issues are illustrated in our findings that anatomical connectivity showed only one area of significant difference whereas functional connectivity showed decreases in all nodes in people with schizophrenia.

Together, these methodological difficulties argue for a more robust approach. Here we have introduced and applied the NBSm methodology which allows us to define an intermodal node set, composed of brain regions that display altered functional and anatomical connectivity in people with schizophrenia. Further, our method ensures that this group of nodes is validated in a statistically reliable way. The intermodal set defined in our present study provides a robust description of a fundamental set underlying both anatomical and functional imaging.

Recent evidence links fronto-temporal abnormalities (van den Heuvel et al., 2010) to symptoms that accompany the disease (Raij et al., 2009, Rotarska-Jagiela et al., 2010), particularly auditory and verbal hallucinations (Jardri et al., 2011, Spencer et al., 2009). Both our regional findings and the identified intermodal set are consistent with this notion and support the theory that schizophrenia is accompanied by abnormalities in bottom-up circuits involving sensory regions such as the left insula, left middle temporal, right cuneus, and left lingual gyrus. Alterations to these regions have been suggested to result in dysfunction in multisensory processing and integration (Williams et al., 2010) and might also play a role in discriminating self-generated internal processes and real externally-generated sensory information (Wylie and Tregellas, 2010).

The presence of frontal cortices in our findings is also consistent with fronto-striatal and fronto-parietal abnormalities identified in paradigms of cognitive function in schizophrenia. For example, fronto-striatal networks are thought to mediate the poor executive functioning typically observed in people with schizophrenia (Camchong et al., 2006, Koch et al., 2008). Abnormalities in this circuit have in fact been proposed as promising biological markers of the disease (Ettinger et al., 2012, Fusar-Poli et al., 2011). Furthermore, fronto-parietal attention and memory network abnormalities are thought to mediate the attention and memory deficits characteristic of schizophrenia (Bassett et al., 2009, Weiss et al., 2011). Such fronto-parietal networks might further mediate complex cognitive processes such as insight (Antonius et al., 2011).

From a network perspective, our findings are particularly relevant when compared to studies of network efficiency and organization. A recent study has described the human connectome as having a core of highly connected hubs, also known as a “rich-club” (van den Heuvel and Sporns, 2011). A rich-club architecture contains a core of hubs that are densely connected among themselves rather than being densely connected to non-hub nodes. This organization may provide resilience against network failure, based on the redundant connectivity of hub nodes. Van den Heuvel and Sporns identified 12 densely connected hub regions including right and left superior parietal, right and left precuneus, right and left superior frontal cortex, right and left putamen, right and left hippocampus, and right and left thalamus. In our study, we also identified the right and left precuneus and right superior parietal regions as members of the intermodal set; i.e. regions with anatomical and functional connectivity significantly different in people with schizophrenia. One could speculate that these common abnormalities could result in a fundamental disruption in the network's ability to recover from intense demands such as biological or psychological stressors.

We have shown NBSm to offer distinct promise as a methodology, but caution is warranted in interpreting our specific findings with respect to the underlying connectomics of schizophrenia. While graph theory is a powerful tool, its metrics can be biased and sensitive to shortcomings and variability in the underlying data and/or data processing techniques. This has been examined in-depth in the literature (Fornito et al., 2013) but warrants discussion of specific considerations. Graph theory relies on accurate and reliable definition of nodes and edges, with edges having the strength of connectivity defined based on the value of underlying data. Should the node or edge definition be unreliable, then the composite network measures will also be unreliable. Drakesmith et al. (2015) and Garrison et al. (2015) have shown that simple thresholding of networks, a common practice used in neuroimaging graph theory to find subnetworks of interest, is an unreliable method causing a larger likely variation in the graph statistics compared to non-thresholded metrics such as streamline counts or functional connectivity. Further, Braun et al. (2012) have shown that second order metrics (small-worldness, hierarchy, and assortativity) are more robust than first order metrics (clustering coefficient, path length, efficiency). Garrison et al. suggests measures of sparsity are reliable at defining subnetworks of interest whereas Drakesmith et al. suggests a multi-threshold technique. NBS and our extension NBSm take a similar approach, by avoiding a first order threshold technique, to discover relevant subnetworks based on the statistical difference in edge strength between groups. This procedure avoids an arbitrary threshold but instead uses the significance of the data to determine connectivity. Of course, our method is not without its own potential pitfalls: the method could be subject to compounded error from the combination of multiple graph theoretical analyses as well as methodological issues in the underlying data.

These sources of error are worth discussion and are likely limitations in our study based on the methodology used to collect and process the data. First, use of DTI has been shown to produce elevated false negatives which could alter the subsequent components produced by NBSm (Feigl et al., 2014, Kuhnt et al., 2013). Newer diffusion paradigms and analysis tools are available and have been shown to be more sensitive at detecting crossing tracts as well as local connectivity tracts (Kuhnt et al., 2013, Proix et al., 2016). We did not have motion parameter data for our DTI. While we found no motion group differences in the fMRI data, this does not necessarily mean there was no motion differences in the DTI data. Second, the validity of our findings may be limited by the short fMRI acquisition length of 6 min, as has been shown elsewhere in the literature (Birn et al., 2013). New multiband techniques are now available and have been shown to reduce scanning time and increase sensitivity to detect resting state brain networks (Preibisch et al., 2015). Third, our anatomical connectivity matrix is likely sensitive to varying brain size. This has been shown to be a common finding in those with schizophrenia (Haijma et al., 2013). This is a possible limitation of our findings as the analysis was performed in native space and there was no normalization step performed prior to subsequent analysis. Fourth, our parcellation technique is an anatomical parcellation and makes use of the AAL atlas (Tzourio-Mazoyer et al., 2002). While this method has been shown to be rapid, intuitive, and reliable it is low resolution, and has large variations in node size (Fornito et al., 2013) which can impact on the reproducibility of connectivity patterns (Craddock et al., 2012, Smith et al., 2011). Future work could utilize subject-specific structural imaging driven parcellations. For example, Proix et al. (2016) evaluated the effects of this sort of parcellation scheme using 140 nodes with structural imaging, and found it to be effective for modelling slow resting state network changes, specifically those found in fMRI. This could provide more reproducible connectivity patterns to use with NBSm. Fifth, in our participants with schizophrenia, neuroleptic medications were being used at differing doses. This has been shown to have significant but unpredictable effects on functional connectivity (De Rossi et al., 2015). Future studies should attempt to normalize neuroleptic dosing across participants. Finally, consideration must be given to the impact of changes in functional connectivity over time. Calhoun et al. (2014) have shown changes in functional connectivity over time within a single resting state imaging session. Future work should examine the NBSm component change within a single resting state session to determine if there are multi-modal components that vary within session.

Overall our approach is novel, robust, and uncovers regions of fundamental importance in their contribution to both anatomical and functional dysconnectivity in schizophrenia through the use of an integrated, multimodal analysis. The work confirms a picture of dysconnectivity in regions thought to mediate central brain processes such as memory, insight, and perceptual experiences. The findings provide specific foci for further study in a field dominated by vast and complex findings. Future work will seek to identify intermodal component-based and node-based diagnostics that correlate with positive symptoms, negative symptoms, and cognitive and neuropsychological variables, which together could provide a critical understanding of long-term clinical prognosis, possible targets of therapy, or biomarkers to assess response to therapy.

## Funding

This work was supported by the National Institute of Mental Health [grant numbers R21MH79262 to K.L., MH-060662 to K.L.]; and the General Clinical Research Center [M01 RR00400]; and the Center for Magnetic Resonance Research [BTRR—P41 RR008079, P30 NS057091]; and the Mind Research Network, Albuquerque, NM.
